# Antibiotics Impact the Cytotoxicity and Cytopathic Effect of *Helicobacter pylori* Extracellular Vesicles Against Gastric Cells

**DOI:** 10.3390/ijms262110399

**Published:** 2025-10-26

**Authors:** Paweł Krzyżek, Mateusz Chmielarz, Edyta Bożemska, Agnieszka Opalińska, Mateusz Olbromski, Michał Małaszczuk, Barbara Krzyżanowska, Katarzyna Haczkiewicz-Leśniak, Marzenna Podhorska-Okołów, Piotr Dzięgiel, Beata Sobieszczańska

**Affiliations:** 1 Department of Microbiology, Faculty of Medicine, Wroclaw Medical University, 50-368 Wrocław, Poland; mateusz.chmielarz@student.umw.edu.pl (M.C.); edyta.bozemska@umw.edu.pl (E.B.); michal.malaszczuk@umw.edu.pl (M.M.); barbara.krzyzanowska@umw.edu.pl (B.K.); beata.sobieszczanska@umw.edu.pl (B.S.); 2 Laboratory of Nanostructures, Institute of High Pressure Physics, Polish Academy of Sciences, 01-424 Warsaw, Poland; a.opalinska@labnano.pl; 3 Department of Human Morphology and Embryology, Division of Histology and Embryology, Faculty of Medicine, Wroclaw Medical University, 50-368 Wrocław, Poland; mateusz.olbromski@umw.edu.pl (M.O.); piotr.dziegiel@umw.edu.pl (P.D.); 4 Department of Microbiology, Faculty of Biological Sciences, University of Wroclaw, 51-151 Wrocław, Poland; 5 Division of Ultrastructural Research, Faculty of Medicine, Wroclaw Medical University, 50-368 Wrocław, Poland; katarzyna.haczkiewicz-lesniak@umw.edu.pl (K.H.-L.); marzenna.podhorska-okolow@umw.edu.pl (M.P.-O.); 6 Department of Physiotherapy, University School of Physical Education, 51-612 Wrocław, Poland

**Keywords:** *Helicobacter pylori*, extracellular vesicles, membrane vesicles, antibiotics, subinhibitory antibiotic concentrations, toxins, cytotoxicity, phenotypic changes

## Abstract

*Helicobacter pylori* is a spiral microorganism capable of inducing a range of gastric diseases. Among different virulence determinants produced by this bacterium, VacA and CagA are of critical importance for the development of these conditions. Taking into account the ability to chronically colonize the stomach, drug-resistant strains of this pathogen can be repeatedly exposed to subinhibitory antibiotic concentrations, which in turn may reduce or enhance their extracellular vesicles (EVs)-derived virulence towards gastric cells. With the use of different experimental techniques, we were the first to demonstrate that subinhibitory antibiotic concentrations modify both the cytotoxicity and cytopathic effect induced by EVs of *H. pylori* in gastric cells. The ability to induce vacuolization and the hummingbird phenotype in gastric cells presented an antibiotic-specific pattern. At the highest doses tested, all EV types induced phenotypic changes and cytotoxicity in gastric cells; however, the highest lethal effect was observed for EVs isolated from native (antibiotic-unexposed) cells. This suggests that short-term exposure of *H. pylori* to subinhibitory antibiotic concentrations does not translate into exacerbation of its EVs-dependent virulence. Nevertheless, extensive research in this area is undoubtedly needed to confirm these observations.

## 1. Introduction

*Helicobacter pylori* is a spiral, microaerophilic, Gram-negative bacterium [1]. This microorganism is capable of colonizing the stomach, thanks to its ability to perceive a chemical gradient and move toward the gastric mucosa [2]. *H. pylori* infections most often occur in childhood with clinical symptoms remaining undetected by many years [3]. The presence of *H. pylori* significantly contributes to the physiological and morphological changes in the stomach, which over time can lead to the development of various pathologies of this organ, such as ulceration or carcinogenic processes (adenocarcinomas or mucosa-associated lymphoid tissue lymphomas) [4,5]. Although *H. pylori* is a non-invasive bacterium, its capacity to induce chronic inflammation and systemically disseminate virulence factors may generate numerous extragastric diseases, ranging from metabolic to cardiovascular and neurological disorders [6]. Among different virulence determinants produced by this bacterium, two toxins are of critical importance for the development of the above-mentioned conditions [4,5,6]. 

The first is the vacuolating cytotoxin A (VacA), whose gene is present in all *H. pylori* strains, albeit in different allelic versions [7,8,9]. The s1/m1 and s1/m2 alleles are thought to cause more severe gastric disorders than the s2 allele. VacA is produced as a 140 kDa protoxin that undergoes limited proteolytic cleavage to form an 88 kDa secretory protein composed of two subunits (p33 and p55). VacA is released into the extracellular environment via the type 5 secretion system (T5SS). When it interacts with the host cell, it disrupts signaling processes, depolarizes the membrane, and induces anion-selective efflux of carbonate and organic ions from the cell interior. In this way, VacA facilitates *H. pylori* survival by increasing the amount of nutrients obtained from the gastric mucosa. Changes in cell membrane permeability and an increase in osmotic pressure contribute to the formation of vacuoles in host cells—a phenotype giving this toxin its name. 

The second key virulence factor of *H. pylori* is the cytotoxin-associated gene A (CagA) [10,11,12]. It is estimated that approximately 70% of strains worldwide produce this toxin, including 60% from Western and 95% from East Asian countries. CagA is a 125–145 kDa oncoprotein injected into host cells via the type 4 secretion system (T4SS). In the cytoplasm of effector cells, CagA undergoes phosphorylation, modulating their signaling and inducing a number of characteristics typical of cancer cells, such as cell death resistance, invasiveness, and genetic instability. Furthermore, CagA disrupts the cytoskeleton of host cells, which consequently contributes to their elongation and increased motility. This phenomenon is referred to as the “hummingbird phenotype” and is the most characteristic pathological change induced by this toxin.

Microorganisms have evolved a variety of single- or two-step secretion systems responsible for the transport of effector proteins into host cells [13,14]. However, an alternative secretion system involving the production of extracellular vesicles (EVs) has been postulated [15]. EVs possess the characteristics of nanoscale vectors, constituting a cocktail of bioactive macromolecules (lipids, proteins or nucleic acids) protected by biological membranes [16]. In this way, microbial components enclosed within the EV lumen are sheltered from enzymatic decomposition or degradation by extreme values of pH or redox potential. Additionally, EVs enable the simultaneous delivery of various virulence factors, allowing for the induction of synergistic pathological effects [17]. Therefore, EVs are considered a crucial element of intercellular pathogen-host interactions [18]. Selective transport of toxins and other lytic factors into host cells via EVs has been demonstrated for many microbial species [17,19]. Although *H. pylori* typically delivers VacA and CagA toxins to host cells using T5SS and T4SS, respectively, the vesiculation process is currently considered to be highly important in the transfer of these virulence factors [20,21,22,23]. 

The pathological effect of VacA and CagA toxins from *H. pylori* on gastric cells is well known and described [1,4,5]. On the other hand, studies on the transfer of these toxins via EVs represent a new research avenue that may deepen the understanding of phenomena underlying the development of diseases caused by this bacterium [22]. As demonstrated for various microbial species, antibiotic therapy may significantly impact the secretion and toxicity of microbial EVs against host cells [24,25,26,27,28,29]. Knowledge regarding the effects of antimicrobial substances on EVs produced by *H. pylori* is however currently in its infancy. To date, only three articles focusing on this topic have been published [30,31,32]. Two of these described the ability of *H. pylori* EVs to induce tolerance to the tested antimicrobials [30,31], while the third, conducted by our team, determined the effect of selected antibiotics on the physicochemical parameters and fatty acid profile of EVs from this bacterium [32]. 

**Figure 1:**
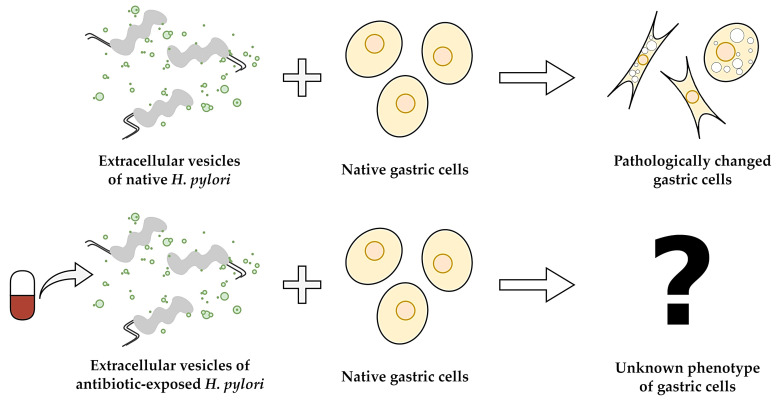
A schematic diagram showing the main objective of the current research, aimed at determining the effect of subinhibitory antibiotic concentrations on the cytotoxicity and cytopathic effect of *H. pylori* EVs against gastric cells.

Building on our previous research and aiming to address the knowledge gap, this article aims to assess the effect of antibiotics on the toxicity of *H. pylori* EVs towards gastric cells (Figure 1). Using a range of experimental techniques, we were the first to demonstrate that antibiotics modify the cytopathic effect induced by EVs of *H. pylori* in gastric cells.

## 2. Results

For our current studies on the cytotoxicity and cytopathic effect of EVs toward gastric cells, we selected the clinical *H. pylori* 3CML strain. This choice was dictated by two factors. Firstly, this strain is multidrug-resistant (resistant to clarithromycin [CLR], metronidazole [MTZ], and levofloxacin [LEV]) [33] and therefore provides an excellent model for testing the effect of subinhibitory (clinically ineffective) antibiotic concentrations on the activity of EVs against gastric cells. Secondly, among all the characterized *H. pylori* strains from our collection, this isolate is equipped with one of the highest adaptive properties under antibiotic pressure, as evidenced by the EV secretion, biofilm production, and coccoid transformation [33,34]. At the same time, from our strains with the highest adaptive capacities, *H. pylori* 3CML presents the highest level of EV production, both with and without antibiotic exposure [32].

**Figure 2:**
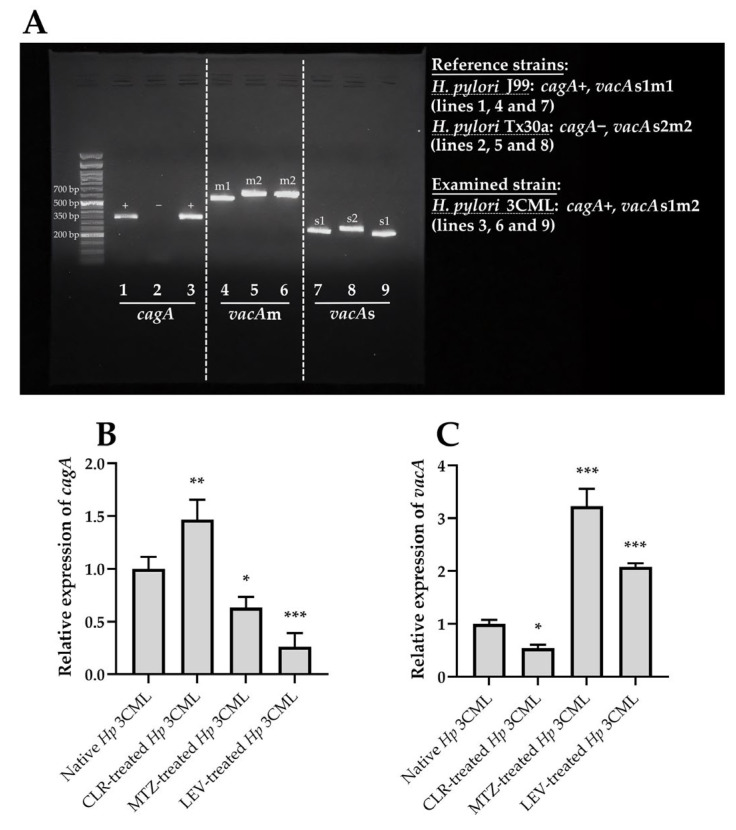
Genotypic analysis of *H. pylori* 3CML and the expression of cytotoxin genes during its antibiotic exposure. (**A**) Determination of the presence of *cagA* and classification of the allelic version of *vacA* in the clinical *H. pylori* 3CML strain. Reference *H. pylori* J99 and Tx30a strains were used as controls (*cagA*+, *vacA* s1m1 and *cagA*-, *vacA* s2m2, respectively). Analysis of (**B**) *cagA* and (**C**) *vacA* expression in native bacteria (unexposed to antibiotics) or treated with ¼× MIC of one of the antibiotics: clarithromycin (CLR), metronidazole (MTZ), or levofloxacin (LEV). The expression of cytotoxin genes was normalized to the expression of the reference *16S rRNA* gene. *, ** and *** indicates statistical difference of *p* < 0.05, *p* < 0.01 and *p* < 0.001, respectively.

Our experiments began by analyzing the toxin profile of *H. pylori* 3CML. We demonstrated that this strain is capable of producing the CagA toxin (*cagA*+) and possesses one of the more toxic variants of VacA (*vacA*s1m2) (Figure 2A). Next, using reverse transcription-quantitative polymerase chain reaction (RT-qPCR), we determined how exposure to subinhibitory concentrations of antibiotics affects the expression of genes encoding these two toxins. We observed an interesting phenomenon, consisting of an increased *cagA* expression and decreased *vacA* expression in bacterial cells exposed to CLR, whereas the opposite was noticed for MTZ or LEV (Figure 2B,C). This suggests that subinhibitory concentrations of classically used antibiotics can significantly modulate the toxigenicity of this pathogen. 

**Figure 3:**
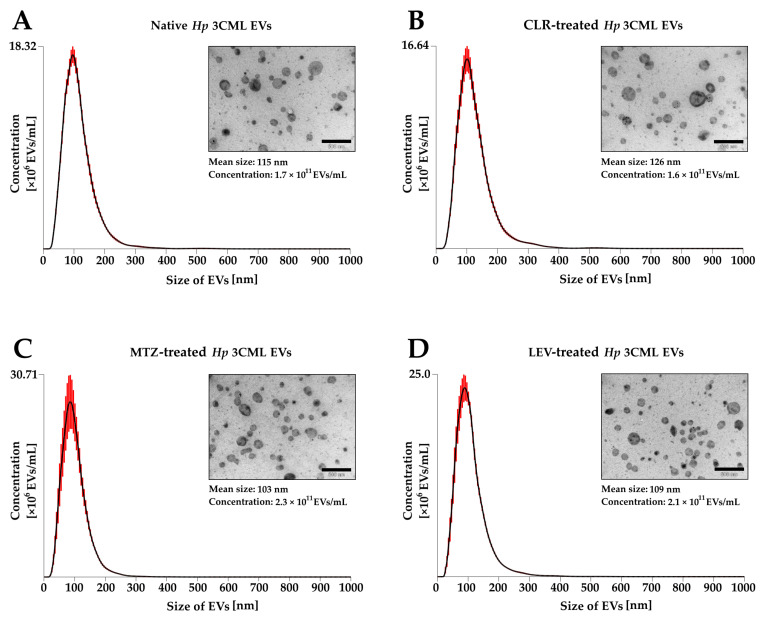
Assessment of biological parameters of extracellular vesicles (EVs) isolated from *H. pylori* 3CML cultured under antibiotic stress. Bacteria were incubated in BHI + 10% FCS^-EVs^ for 3 days at 37 °C under microaerophilic conditions and were shaked at 100 rpm. Bacteria were (**A**) not exposed to any antibiotics (native) or treated with ¼× MIC of one of the antibiotics: (**B**) clarithromycin (CLR), (**C**) metronidazole (MTZ), or (**D**) levofloxacin (LEV). The concentration, mean size, and size distribution of EVs were measured using nanoparticle tracking analysis, while their ultrastructure was imaged using transmission electron microscopy. Scale bars, 500 nm.

Knowing the profile of toxins and the effect of antibiotics on their expression, we managed to isolate and characterize EVs of *H. pylori* 3CML according to our established methodology of culturing bacteria in conditions devoid of animal-derived EVs in serum applied to liquid culture media [32]. Firstly, we demonstrated that the subinhibitory antibiotic concentrations did not significantly affect the growth of *H. pylori* 3CML in these conditions (Figure S1). Secondly, based on nanoparticle tracking analysis (NTA), we confirmed a high EV secretion capacity for this strain. The concentration and the mean size of EVs were in the range of 1.6–2.3 × 10^11^ EVs/mL and 103–126 nm, respectively (Figure 3). Interestingly, we observed a pattern of increasing EV dimensions with decreasing total concentration (1.6 × 10^11^ EVs/mL and 126 nm in CLR-treated bacteria and 2.3 × 10^11^ EVs/mL and 103 nm for those exposed to MTZ). The presence of EVs in the samples and the absence of contaminating structures (e.g., flagella) was additionally confirmed by us using transmission electron microscopy (TEM) (Figure 3).

**Figure 4:**
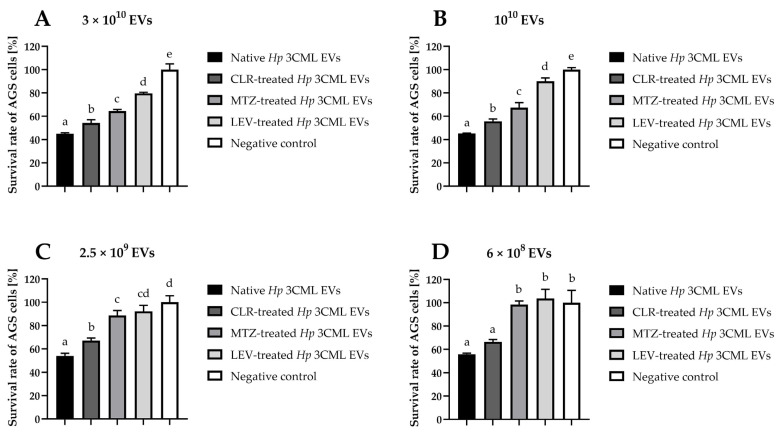
Cytotoxicity of extracellular vesicles (EVs) from *H. pylori* 3CML against the AGS cell line. EVs are coming from native (antibiotic non-exposed) cells or those treated with ¼× MIC of one of the antibiotics: clarithromycin (CLR), metronidazole (MTZ), or levofloxacin (LEV). The negative control constituted a PBS-resuspended sample from BHI + 10% FCS^-EVs^ without bacteria, which was subjected to the similar EVs isolation steps as other samples. AGS cells were co-incubated with (**A**) 3 × 10^10^, (**B**) 10^10^, (**C**) 2.5 × 10^9^, or (**D**) 6 × 10^8^ of *H. pylori* 3CML EVs. The cytotoxicity level of these structures was assessed using the MTT assay. Values with different letters in columns are statistically different (*p* < 0.05).

After characterizing the strain’s toxin profile and isolating its EVs, we proceeded to the first stage of the main experimental phase by determining the cytotoxicity of these structures towards AGS cells. As expected, we observed a dose-dependent toxic effect (*p* < 0.05) (Figure 4). Among the EV variants tested, those derived from native bacteria (unexposed to antibiotics) or CLR-treated ones exhibited a higher level of cytotoxicity than EVs isolated from the strain treated with MTZ or LEV (*p* < 0.05). Regardless of the EV variant used, however, doses of 3 × 10^10^ and 10^10^ caused a statistically significant decrease in AGS viability in all samples (down to 45–79.6% and 45.2–90%, respectively; Figure 4A,B). At the lowest dose (6 × 10^8^), only EVs from native and CLR-treated bacteria maintained their cytotoxicity (survival rates of 55.9% and 66.4%, respectively; Figure 4D).

**Figure 5:**
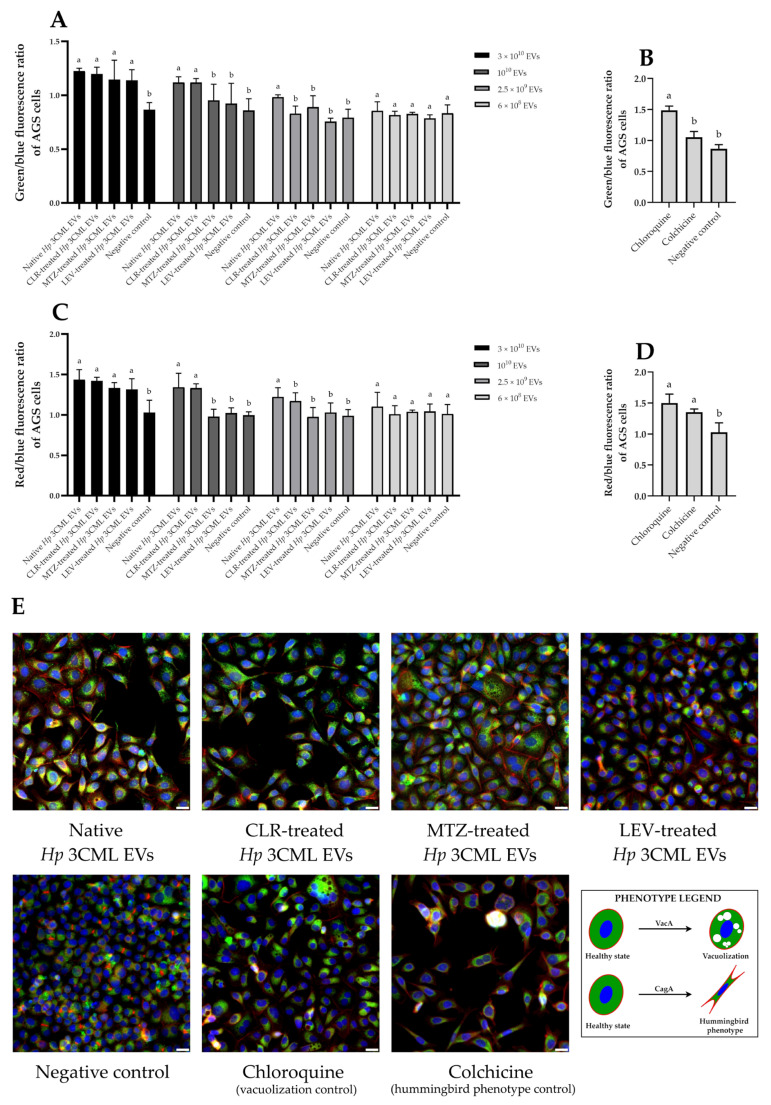
Phenotypic changes in the AGS cell line after 24 h exposure to extracellular vesicles (EVs) from *H. pylori* 3CML measured by fluorescence microscopy. EVs are derived from native (antibiotic non-exposed) cells or those treated with ¼× MIC of one of the antibiotics: clarithromycin (CLR), metronidazole (MTZ), or levofloxacin (LEV). The negative control constituted a PBS-resuspended sample from BHI + 10% FCS^-EVs^ without bacteria, which was subjected to the similar EVs isolation steps as other samples. Chloroquine (0.1 µM) and colchicine (10 µM) were used as positive controls for vacuolization and hummingbird phenotype, respectively. AGS cells were stained with DAPI (blue), FM 1-43 (green), and phalloidin-conjugated Alexa Fluor 647 (red) to visualize the cell nucleus, cell membrane, and cytoskeleton, respectively. Fluorescence intensity of the cell membrane (**A**,**B**) or cytoskeleton (**C**,**D**) of AGS cells after exposure to a concentration gradient of *H. pylori* 3CML EVs or control substances. Values with different letters in columns are statistically different (*p* < 0.05). (**E**) Representative fluorescence images of AGS cells treated with 3 × 10^10^ of EVs or control samples. Scale bars, 50 µm.

To further increase our understanding of the effects of the studied *H. pylori* EVs on gastric cell physiology, we performed a series of fluorescent microscopic observations and selective staining of cellular components (Figure 5). Analyzing the post-microscopic data, we observed that EVs used at the highest dose (3 × 10^10^) induced a significant increase in the fluorescence of the analyzed structures (Figure 5A,C). This phenomenon also occurred when using reference substances (chloroquine—a vacuolization control [35], and colchicine—a hummingbird phenotype control [36]; Figure 5B,D), but not with lower EV doses (Figure 5A,C). Linking the facts together, we hypothesized that EVs at a dose of 3 × 10^10^ might induce changes in the permeability of AGS cell membranes and stimulate an increase in the uptake of fluorescent dyes from the extracellular environment. Because changes in host membrane permeability are characteristic of both VacA and CagA [37,38], we closely examined the phenotypic changes in AGS cells exposed to 3 × 10^10^ EVs of *H. pylori* 3CML. Indeed, we confirmed that many of them showed changes visually resembling vacuolation and/or elongation (Figure 5E).

**Figure 6:**
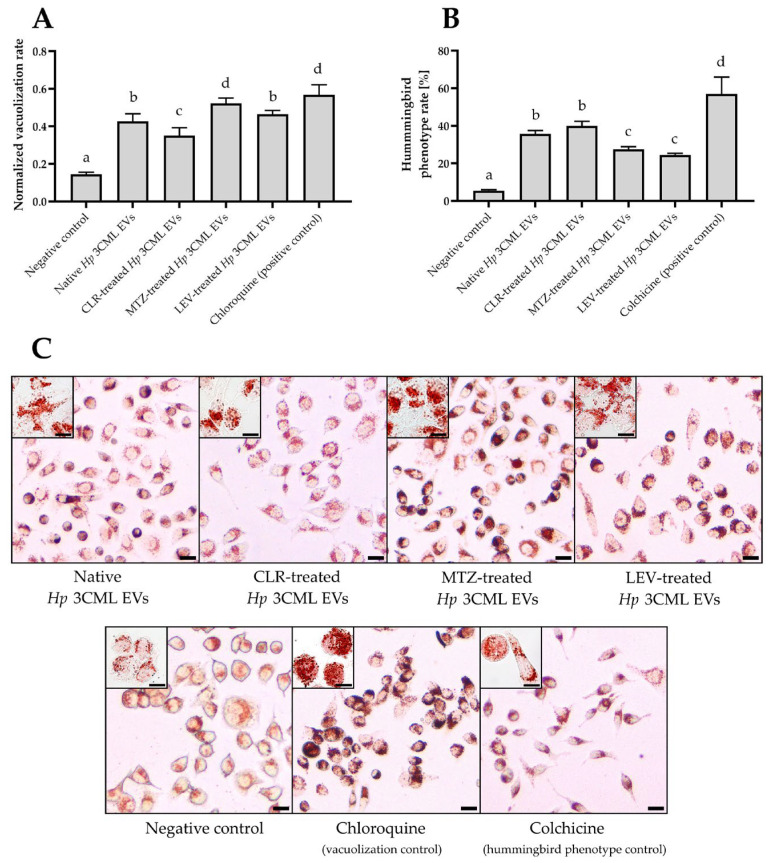
Phenotypic changes in the AGS cell line after 24 h exposure to extracellular vesicles (EVs) from *H. pylori* 3CML measured by light microscopy and a neutral red staining. EVs are coming from native (antibiotic non-exposed) cells or those treated with ¼× MIC of one of the antibiotics: clarithromycin (CLR), metronidazole (MTZ), or levofloxacin (LEV). The negative control constituted a PBS-resuspended sample from BHI + 10% FCS^-EVs^ without bacteria, which was subjected to the similar EVs isolation steps as other samples. Chloroquine (0.1 µM) and colchicine (10 µM) were used as positive controls for vacuolization and hummingbird phenotype, respectively. (**A**) Degree of vacuolization or (**B**) degree of elongation (the hummingbird phenotype) of AGS cells after exposure to 3 × 10^10^ of *H. pylori* 3CML EVs or control substances. Values with different letters in columns are statistically different (*p* < 0.05). (**C**) Representative images of AGS cells treated with 3 × 10^10^ of EVs or control samples. Scale bars, 10 µm.

In the third stage of the main experimental phase, we decided to confirm the observations made with fluorescence microscopy. To achieve this, we used light microscopy and a neutral red staining—a dye targeting vacuoles [39] (Figure 6). This type of monochromatic image analysis facilitated the easier interpretation of the results. In all samples, we noted an augmented absorption of neutral red (Figure 6A,C). Simultaneously, we observed that AGS cells exposed to EVs coming from MTZ- or LEV-treated *H. pylori* 3CML had higher levels of absorbed dye (0.52 and 0.46, respectively) than after exposure to EVs derived from native bacteria (0.43) or those treated with CLR (0.35). Additionally, based on the shape, we categorized AGS cells into physiological and elongated ones, and confirmed the ability of the analyzed EVs to induce the hummingbird phenotype (Figure 6B,C). In this context, the highest level of cellular elongation was observed for EVs of CLR-treated bacteria (40%), while the lowest for EVs of LEV-treated ones (24.5%). The data of the current experimental stage correlate well with toxins’ gene expression in *H. pylori* 3CML (Figure 2B,C), suggesting that EVs secreted by this strain contain toxins whose levels and ability to induce cytopathic effects in AGS cells are modulated by subinhibitory antibiotic concentrations.

**Figure 7:**
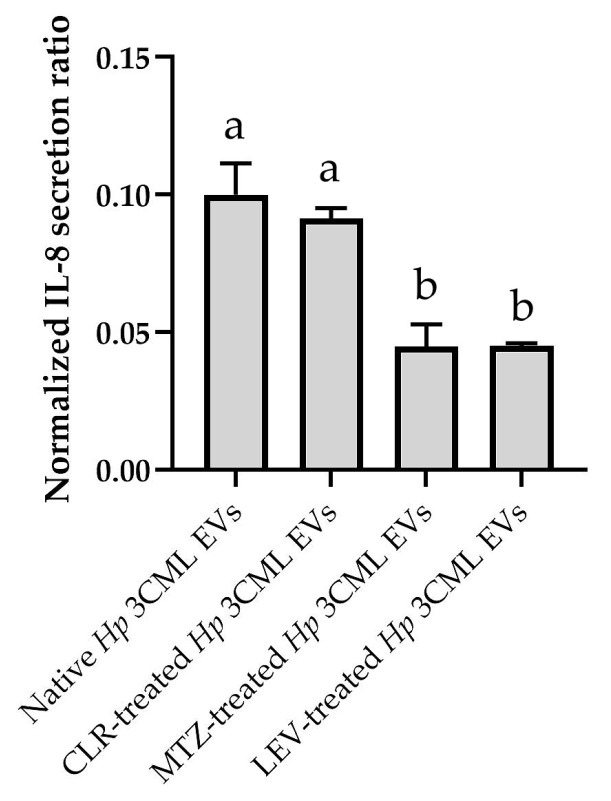
Induction of a proinflammatory response in the AGS cell line after 24 h exposure to 3 × 10^10^ of extracellular vesicles (EVs) from *H. pylori* 3CML. EVs are coming from native (antibiotic non-exposed) cells or those treated with ¼× MIC of one of the antibiotics: clarithromycin (CLR), metronidazole (MTZ), or levofloxacin (LEV). A commercial IL-8 enzyme-linked immunosorbent assay kit was used to determine the level of this cytokine in the supernatant of AGS cells. The obtained data were normalized to the absorbance values acquired in the MTT assay. In each case, the values obtained for the tested samples were subtracted from the negative control results. Values with different letters in columns are statistically different (*p* < 0.05).

In the final step, we aimed to establish whether the EVs used were capable of stimulating proinflammatory cytokines in the AGS line and whether antibiotics could influence this effect. Indeed, we confirmed an increase in IL-8 levels in the post-culture supernatant of AGS cells exposed to all EV variants (Figure 7). Furthermore, we observed a strong correlation between the MTT-based viability analyses (Figure 4) and the present data of IL-8 secretion levels (Figure 7). In this regard, the highest proinflammatory induction was observed for EVs isolated from native *H. pylori* 3CML cells, followed by those exposed to CLR, then both MTZ and LEV.

## 3. Discussion

The ineffectiveness of antimicrobial therapies is a long-standing challenge for the healthcare sector. The latest and most comprehensive study analyzing global mortality over the past three decades revealed this to be one of the greatest problems [40]. In 2021 alone, nearly 5 million deaths were attributed to infections caused by antibiotic-resistant bacteria, and this number is expected to rise to 8 million by 2050. On a worldwide scale, the economic burden of antibiotic resistance costs hundreds of trillions of dollars annually [41]. The issue of drug resistance and its associated costs is also very current for infections caused by *H. pylori*. A recent international, multicenter study showed that antibiotic resistance of *H. pylori* to the most commonly used and effective antimicrobials (CLR, MTZ, and LEV) is increasing [42]. The prevalence of antibiotic resistance in this pathogen varies across the world, although it is significantly higher in developing countries and is greatly fueled by the overuse and misuse of antimicrobial drugs [43]. In this context, a strong correlation between the level of outpatient consumption of macrolides, fluoroquinolones, and nitroimidazoles in the treatment of other microbial diseases and the development of *H. pylori* resistance is noticed [42,44]. Furthermore, we believe that this phenomenon may play a significant role not only in generating *H. pylori* strains resistant to a given class of antimicrobial compounds, but also in modulating their virulence traits once such resistance has developed. 

Antibiotics induce a number of changes in the physiology and virulence of microorganisms [45,46], primarily when used at subinhibitory concentrations [47]. These modifications can affect both their survival capacity (e.g., biofilm formation) and toxicity (e.g., invasiveness, production of toxins or lytic enzymes) [45,46,47]. Although the impact of antibiotics on microbial vesiculation remails a relatively unexplored research area, there are different scientific reports demonstrating the effect of antimicrobials on the quantity and/or toxicity of EVs towards host cells [24,25,26,27,28,29]. In line with this, we believe that antibiotic-resistant *H. pylori* constitutes an excellent research model for determining the significance of this phenomenon. Because of the ability to chronically colonize the stomach [4], drug-resistant strains of this pathogen can be repeatedly exposed to subinhibitory antibiotic concentrations, which in turn may reduce or enhance the cytotoxicity of their EVs towards gastric cells (Figure 1). 

In the current study, we determined the effect of EVs derived from a multidrug-resistant *H. pylori* strain exposed to subinhibitory concentrations of CLR, MTZ or LEV on a number of physiological characteristics of gastric cells. The methodology of the current EV isolation was consistent with that developed by us previously [32]. For this purpose, we used bacteriological culture in serum devoid of animal-borne EVs and estimated precisely the concentration of isolated EVs by NTA. This approach is consistent with good practices recommended by others [48] and certainly contributed to the reliability of our data. According to the data of Zavan et al. [49], a 3-day incubation of *H. pylori* contributes to the highest production of EVs and the greatest abundancy of virulence factors carried in these structures, therefore we limited our analysis only to EVs coming from this growth stage.

In the first stage of the experiments, we observed that the cytotoxicity of *H. pylori* EVs toward gastric cells increased with increasing doses of these structures, which is convergent with results from other studies [50,51]. Interestingly, we observed that the highest level of toxicity was observed for EVs derived from native bacteria (untreated with antibiotics), then exposed to CLR, and finally to MTZ or LEV. At the same time, the data from the cytotoxicity assays correlated well with our observations on the capacity of these structures to induce IL-8 in gastric cells. Although the exact cause of this phenomenon is unknown, literature data allowed us to explain its source. Antibiotics are able to stimulate microbial vesiculation, while the intensity of this process depends largely on the molecular mechanism of their action [52]. Antimicrobials that target nucleic acids, including MTZ or LEV, are potent inducers of vesiculation [24,53,54], whereas substances that inhibit translation (e.g., CLR) do not cause significant changes in the EV synthesis [24]. Our team made similar observations in the previous study on *H. pylori* [32]. Therefore, we believe that increased EV production during exposure of *H. pylori* to MTZ or LEV may contribute to a stoichiometric reduction in the virulence factors deposited in EVs and a decrease in their cytotoxicity. Unlike the previous two antibiotics, exposure of *H. pylori* to CLR does not significantly affect vesiculation and thus the level of cytotoxicity of EVs derived from these conditions is similar with EVs of native bacteria.

Considering that CagA and VacA carried in *H. pylori* EVs may be responsible for the cytotoxic effect [20,21,22,23], we decided to determine the phenotypic changes occurring in gastric cells exposed to these structures. We noticed that at the highest doses, all types of *H. pylori* EVs were capable of inducing both cell elongation (a hummingbird phenotype) and vacuolization. The magnitude of this phenomenon was interrelated with the toxins’ gene expression levels, confirming our suspicion that these two toxins are of the highest importance in the cytotoxicity of *H. pylori* EVs. Nevertheless, we are aware that EVs produced by *H. pylori* may carry dozens of different virulence factors [49,55,56]. These additional virulence determinants include primarily metabolic enzymes, urease subunits, and adhesins (lipopolysaccharide subunits and outer membrane proteins), which are responsible for nutrient acquisition, neutralization of the acidic environment, and uptake of EVs into host cells, respectively. Therefore, although it appears that the cytotoxicity of the examined EVs was substantially associated with the action of VacA and CagA toxins, the potential existence of synergistic action of several virulence factors cannot be definitively excluded. In line with this, we plan to expand our studies in the future to include proteomic analyses of *H. pylori* EVs and to determine how exposure of *H. pylori* to subinhibitory antibiotic concentrations affects the cytotoxicity and cytopathic effect of whole bacterial cells against gastric cells. All of the above will help us to understand better the intricate interactions in the antibiotics-*H. pylori*-host axis.

## 4. Materials and Methods

### 4.1. Microbiological Experiments

#### 4.1.1. Storage and Revival of Bacteria

The methodology for storing and maintaining *H. pylori* was in accordance with our established procedure [33,34]. A multidrug-resistant clinical *H. pylori* 3CML strain, belonging to the collection of the Department of Microbiology at Wroclaw Medical University, was chosen for the present study. The strain was stored at −80 °C in Tryptic Soy Broth (TSB; Oxoid, Dardilly, France) supplemented with 30% glycerol until use in experiments. To reactivate the strain, the frozen stock of bacteria was plated on Columbia agar (Difco, Lublin, Poland) enriched with 10% horse blood (GRASO, Starogard Gdanski, Poland) and incubated for 3 days at 37 °C under microaerophilic conditions (Genbox microaer kits, BioMerieux, Marcy l’Étoile, France). Following this, the strain was subcultured onto fresh agar plates and incubated under the same conditions for an additional 3 days. Then, the strain was proceeded to subsequent experimental stages.

#### 4.1.2. Genetic Analyses

The analysis of *H. pylori* 3CML toxin profile was performed using the PCR method and selective primers designed in other study [57]. Genetic material obtained from agar-based bacterial cultures was subjected to DNA isolation using the Syngen DNA Mini Kit (Syngen Biotech, Wrocław, Poland), according to the manufacturer’s instructions. Details of the target gene, amplicon size, primer names and sequences are provided in Table 1. For each PCR reaction, 1–2 μg of DNA was added to a 25-μL PBS-based reaction mixture containing 1 µM of each primer (forward and reverse; Oligo.pl, IBB, Warsaw, Poland), 1 mM MgCl_2_ (ThermoFisher Scientific, Waltham, MA, USA), 1.25 U of a DreamTaq DNA polymerase (ThermoFisher Scientific, Waltham, MA, USA), and 200 μM of each dNTP (Oligo.pl, IBB, Warsaw, Poland). The PCR amplification protocol was performed using a PTC-200 Thermal Cycler (Bio-Rad Laboratories, Hercules, CA, USA) and included an initial denaturation at 95 °C for 3 min, followed by 35 cycles of denaturation at 95 °C for 30 s, annealing for 30 s (see Table 1 for specific temperatures), and extension at 72 °C for 30 s. A final polymerization step was carried out at 72 °C for 5 min. PCR products were separated by electrophoresis on a 1.8% agarose gel. Bands were visualized with ethidium bromide staining and analyzed using the Gel-Doc 2000 software (Bio-Rad Laboratories, Hercules, CA, USA), and their size was estimated using a FastGene 50 bp DNA Marker (50 bp–1500 bp; NIPPON Genetics, Düren, Germany). Genetic material from *H. pylori* J99 and Tx30a cultures was used as a control when estimating a toxigenic profile of the analyzed *H. pylori* strain (*cagA*+, *vacA* s1m1 and *cagA*-, *vacA* s2m2, respectively). 

The assessment of the effect of antibiotics on the expression of *H. pylori* 3CML toxin genes was performed using RT-qPCR and primers designed earlier by others (Table 2) [58,59]. Bacteria were grown in 24-well titration plates (Bionovo, Legnica, Poland) filled with Brain-Heart Infusion broth (BHI; Oxoid) and 10% EV-depleted fetal calf serum (FCS^-EVs^; Gibco, Paisley, Scotland, UK). Each well of the 24-well titration plate was loaded with 1.5 mL of the culture medium, a bacterial suspension (10^8^ CFU/mL), and ¼× minimal inhibitory concentration (¼× MIC) of one of the tested antibiotics (CLR, MTZ or LEV; all from Merck, St. Louis, MO, USA). Wells containing only the bacterial suspension (without antibiotic) were used as a positive control. The plates were incubated for 3 days at 37 °C under microaerophilic conditions with shaking at 100 rpm. After this period, the bacterial cells were centrifuged, the supernatant was discarded, and the remaining pellet was used for the isolation of genetic material. Total RNA was extracted using the RNeasy Mini Kit (Qiagen, Hilden, Germany) and complementary DNA (cDNA) was synthesized using the iScript Reverse Transcription Supermix (Bio-Rad, Hercules, CA, USA). RNA concentration and purity were assessed with a NanoDrop ND-1000 spectrophotometer (ThermoFisher Scientific, Waltham, MA, USA), checking the 260/280 and 260/230 absorbance ratios to ensure there was no contamination from proteins or solvents. RT-qPCR was performed in 20 μL reaction volumes using the SsoAdvanced Universal SYBR Green Supermix (Bio-Rad, Hercules, CA, USA) on an Applied Biosystems 7500 Fast Real-Time PCR System (Foster City, CA, USA). The thermal cycling conditions were as follows: polymerase activation at 95 °C for 15 min, then 45 cycles of denaturation at 95 °C for 15 s, annealing at 60 °C for 20 s, and extension at 72 °C for 20 s. Gene expression levels were normalized to a reference gene (*16S rRNA*), and the relative mRNA expression was calculated using the ΔΔCt method. Each reaction was run in triplicate (*n* = 3).

#### 4.1.3. Isolation of EVs and Their Characterization

The methodology for production and isolation of *H. pylori* EVs was consistent with that established in our previous research [32]. 

Bacteria were grown in 24-well titration plates filled with BHI and 10% FCS^-EVs^. Each well of the 24-well titration plate was loaded with 1.5 mL of the culture medium, a bacterial suspension (10^8^ CFU/mL), and ¼× MIC of one of the tested antibiotics (CLR, MTZ or LEV). Wells containing only the bacterial suspension (without antibiotic) and only culture medium (without bacteria) were used as positive and negative controls, respectively. The plates were incubated for 3 days at 37 °C under microaerophilic conditions with shaking at 100 rpm. The optical density of bacteria was evaluated spectrophotometrically using an Asys UVM 340 microplate reader (Biochrom Ltd., Cambridge, UK) at OD_600_ each day. The values of negative controls (culture medium without bacteria) were subtracted from the measurements of the experimental samples. Three biological repetitions of this analysis were performed (*n* = 3). After a 3-day culture, the samples were directed for the EV isolation procedure. 

EVs were isolated using a polymer-based method with the Total Exosome Isolation Kit (from cell culture media; ThermoFisher Scientific, Waltham, MA, USA), following the manufacturer’s instructions. In brief, bacterial cultures were first centrifuged at 2000× *g* for 30 min and the resulting supernatant was filtered through a 0.45 µm cellulose acetate filter (Corning, New York, NY, USA). The filtrate was then combined with the isolation reagent at a 2:1 ratio, vortexed, and incubated overnight at 4 °C. Subsequently, samples were centrifuged at 10,000× *g* for 60 min, the supernatant was discarded, and the EV-containing pellet was resuspended in PBS (Merck, St. Louis, MO, USA). To ensure the absence of bacterial contamination, 10 µL of the EV solution was plated on Columbia agar supplemented with 10% horse blood and incubated for 7 days at 37 °C under microaerophilic conditions. EV preparations were stored at −80 °C until further analysis. 

The concentration, mean size, and size distribution of EVs were assessed using NTA with the NanoSight NS500 system (Malvern Instruments, Malvern, UK), employing a 405 nm blue diode laser as the light source. Measurements were carried out at an ambient temperature of approx. 23 °C. Each biological sample was analyzed through nine independent runs and the average of these values was reported. As particle concentrations exceeded the detection range, samples were diluted with PBS and the dilution factor was incorporated into the final EV concentration calculations. 

The ultrastructure of EVs was evaluated using TEM. EV samples suspended in PBS were applied to Formvar-carbon-coated copper grids (200 mesh, Ted Pella, Redding, CA, USA) and fixed with 2% paraformaldehyde (ThermoFisher Scientific, Waltham, MA, USA), and 2.5% glutaraldehyde (Serva Electrophoresis, Heidelberg, Germany). Grids were then counterstained with Uranyless (Electron Microscopy Sciences, Hatfield, PA, USA), followed by 3% lead citrate (Electron Microscopy Sciences, Hatfield, PA, USA). Finally, they were embedded in 0.13% methyl cellulose (viscosity 25 cP; Merck, St. Louis, MO, USA) and examined using a JEM-1011 TEM (Jeol, Tokyo, Japan) operated at an accelerating voltage of 80 kV.

### 4.2. Cell Line Experiments

#### 4.2.1. Storage and Revival of the Cell Line

The methodology for storing and maintaining cell line cultures was in accordance with the procedure used by us previously [60], with minor modifications. Human Gastric Adenocarcinoma AGS cells (CRL-1739; ATCC, Manassas, VA, USA) were kept in liquid nitrogen in cryopreservation medium containing DMEM medium (ThermoFisher Scientific, Waltham, MA, USA), 10% fetal bovine serum (FBS; Corning, New York, NY, USA), 1% penicillin-streptomycin (100 U/mL–100 µg/mL; Corning, New York, NY, USA), and an addition of 10% dimethyl sulfoxide (DMSO; Merck, St. Louis, MO, USA). Cells were frozen in a controlled manner to achieve a cooling rate of approximately −1 °C/min and then were transferred to liquid nitrogen (−196 °C). To revive them, the cryotube with the frozen cells was rapidly thawed in a water bath at 37 °C for approx. 1–2 min. The content was transferred to a conical Falcon tube (Corning, New York, NY, USA) containing 10 mL of pre-warmed culture medium without DMSO, centrifuged (1000 rpm, 5 min), and then the supernatant was removed. The cells were resuspended in the fresh medium and seeded into a T75 flask (Sarstedt, Nümbrecht, Germany) with the appropriate amount of the medium.

AGS cells were routinely cultured in DMEM medium supplemented with 10% FBS and 1% penicillin-streptomycin in an incubator maintaining at 37 °C, 5% CO_2_, and high humidity (Heracell 150i, ThermoFisher Scientific, Waltham, MA, USA). After reaching an appropriate confluence of 80–90%, the cells were detached from a T75 flask using 0.05% trypsin-EDTA (ThermoFisher Scientific, Waltham, MA, USA) and seeded into 24-well titration plates (TPP Techno Plastic Products, Trasadingen, Switzerland), 96-well titration plates (TPP Techno Plastic Products, Trasadingen, Switzerland) or 8-well cell culture slides (SPL Life Sciences, Pocheon, Republic of Korea), depending on the type of microscopic or functional evaluation performed.

#### 4.2.2. Cytotoxicity

The cytotoxicity of *H. pylori* EVs towards AGS cells was assessed using the MTT assay [51]. AGS cells were seeded at a density of 5 × 10^3^ cells/well into 96-well titration plates containing 100 µL of DMEM enriched with 10% FBS and 1% penicillin-streptomycin. The cells were incubated under standard conditions (37 °C, 5% CO_2_) to allow for their adhesion and achievement of 80–90% confluence. After this, the AGS cells were treated with *H. pylori* EVs derived from bacteria non-exposed to any antibiotic (a native sample) or those treated with ¼× MIC of CLR, MTZ, or LEV. In each case, various doses of *H. pylori* EVs were used: 3 × 10^10^, 10^10^, 2.5 × 10^9^, and 6 × 10^8^. The negative control constituted a PBS-resuspended sample from a BHI broth with 10% FCS^-EVs^ without bacteria, which was subjected to the similar EVs isolation steps as described in Section 4.1.3. The volume of the negative controls was adequate to the volume of each tested sample. For all samples, an incubation continued for 24 h under standard culture conditions. Then, the medium in each case was replaced with 100 µL of fresh medium containing an additional 0.5 mg/mL of MTT (Merck, St. Louis, MO, USA). Plates were incubated for 3 h at 37 °C in the dark. After incubation, the medium was removed and the formazan was dissolved in 100 µL of DMSO. Absorbance was measured at 570 nm using an Asys UVM 340 microplate reader (Biochrom Ltd., Cambridge, UK). Results were expressed as the percentage viability compared to the negative controls. The tests were performed in three biological replications (*n* = 3).

#### 4.2.3. Fluorescent Microscopy Observations

Analysis of phenotypic changes in cell lines using fluorescent microscopy and selective staining of cellular components was performed in line with others [61,62], with some modifications. AGS cells were seeded onto sterile glass slides with a 13 mm diameter (Bionovo, Legnica, Poland) at a density of 5 × 10^4^ cells/well. After this, the glass slides with the AGS cells were placed in wells of 24-well titration plates filled with 100 µL of DMEM enriched with 10% FBS and 1% penicillin-streptomycin, and incubated under standard conditions (37 °C, 5% CO_2_) to achieve 80–90% confluence. Next, the cells were then treated for 24 h with *H. pylori* EVs coming from bacteria non-exposed to any antibiotic (a native sample) or those treated with ¼× MIC of CLR, MTZ, or LEV. In each instance, a gradient of *H. pylori* EVs was applied: 3 × 10^10^, 10^10^, 2.5 × 10^9^, and 6 × 10^8^. The negative control constituted a PBS-resuspended sample from a BHI broth with 10% FCS^-EVs^ without bacteria, which underwent the same EVs isolation procedure as outlined in the “4.1.3. Isolation of EVs and their characterization” section. The volume of the negative samples corresponded to the volume of each tested sample. Additionally, chloroquine (0.1 µM; Merck, St. Louis, MO, USA) and colchicine (10 µM; Merck, St. Louis, MO, USA) were used as positive controls for vacuolization [63] and hummingbird phenotype [36], respectively, the concentrations of which were experimentally established by us during the validation stage of the methodology. After incubation, the medium was removed and the cells were washed three times with pre-warmed PBS. The cells were fixed with 4% buffered formaldehyde (Chempur, Piekary Śląskie, Poland) for 10 min at room temperature and then washed three more times with PBS to remove residual formaldehyde.

After fixing the cells, a staining solution containing 20 µL of PBS and 0.5 µL of each fluorescent dye: DAPI, FM 1-43, and phalloidin-conjugated Alexa Fluor 647 (all from ThermoScientific, Waltham, MA, USA) was added to each well. These dyes were used to visualize the cell nucleus [64], cell membrane (including vacuolization [65,66,67]), and cytoskeleton (including a hummingbird phenotype [68,69]), respectively. The cells were left for a 15 min-incubation in the dark and then were observed using a Carl Zeiss inverted fluorescence microscope (GmbH, Jena, Germany). Post-microscopic analysis of the obtained photographs was performed using the ImageJ software version 1.54j. In order to normalize differences in the cell density between analyzed samples, the fluorescence intensities of FM 1-43 and phalloidin-conjugated Alexa Fluor 647 were normalized toward the fluorescence of DAPI, and the values were presented as ratios of these two [70]. The tests were performed in three biological replications (*n* = 3).

#### 4.2.4. Light Microscopy Observations

Analysis of phenotypic changes in cell lines using light microscopy was performed in line with others [71,72], with some modifications. AGS cells were seeded at a density of 3 × 10^4^ cells/well into 8-well cell culture slides filled with 200 µL of DMEM with 10% FBS and 1% penicillin-streptomycin. The cells were incubated under standard conditions (37 °C, 5% CO_2_) to achieve 80–90% confluence. The AGS cells were then exposed to 3 × 10^10^ of *H. pylori* EVs from bacteria non-exposed to any antibiotic (a native sample) or those treated with ¼× MIC of CLR, MTZ, or LEV. The negative control constituted a PBS-resuspended sample from a BHI broth with 10% FCS^-EVs^ without bacteria, which underwent the same EVs isolation procedure as outlined in Section 4.1.3. Chloroquine (0.1 µM; Merck, St. Louis, MO, USA) and colchicine (10 µM; Merck, St. Louis, MO, USA) were used as positive controls for vacuolization [63] and hummingbird phenotype [36], respectively. Incubation continued for 24 h under standard conditions. After culture, the cells were washed with PBS and incubated for 10 min with neutral red (400 µg/mL). Observations were performed with an Olympus BX50 microscope (Olympus, Tokyo, Japan). Post-microscopic analysis of the obtained photographs was performed using the ImageJ software version 1.54j. The degree of vacuolation was calculated by measuring the intensity of the absorbed red dye [71], the value of which was normalized to the surface area occupied by the observed cells. The hummingbird phenotype was defined when the ratio of the longest to shortest diameters of cells was greater than 2.5 [72]. All cells from the observation fields were counted. For both tested parameters three biological replicates with three technical replicates representing different observation fields were made (*n* = 9).

#### 4.2.5. Stimulation of Proinflammatory Cytokines

A commercial IL-8 enzyme-linked immunosorbent assay kit (Human IL-8 Elisa Kit; ThermoFisher Scientific, Waltham, MA, USA) was used to determine the level of this cytokine in the supernatant of AGS cells [73,74]. IL-8 concentration was measured in culture medium collected from AGS cells exposed for 24 h to 3 × 10^10^ of *H. pylori* EVs (for culture conditions, see Section 4.2.4). Analyses were performed according to the manufacturer’s instructions. The obtained data were normalized to the absorbance values acquired in the MTT assay. In each case, the values obtained for the tested samples were subtracted from the negative control results. The tests were performed in three biological replications (*n* = 3).

### 4.3. Statistical Analysis

Statistical analyses were conducted using GraphPad Prism version 10 (GraphPad Software, San Diego, CA, USA). The Shapiro–Wilk test was applied to assess the normality of data distribution. As the data followed a normal distribution, subsequent analyses were performed using one-way or two-way ANOVA. A *p*-value of less than 0.05 was considered indicative of statistical significance.

## 5. Conclusions

In this study, we demonstrated that antibiotics used at subinhibitory concentrations toward *H. pylori* affect the expression of *cagA* and *vacA* and modulate the cytotoxicity and cytopathic effects of its EVs on gastric cells. These pathological processes induced by EVs of antibiotic-treated bacteria are lower compared to those of EVs from native (antibiotic-unexposed) cells. This suggests that short-term exposure of *H. pylori* to subinhibitory antibiotic concentrations does not translate into exacerbation of its EVs-driven virulence. However, extensive research in this area is undoubtedly needed to confirm these observations.

## Figures and Tables

**Table 1:** Primer sets used for genotyping *H. pylori* by PCR.

Target Gene	Amplicon Size (bp)	Sequences	Annealing Temperature	Reference
*cagA*	349	F: 5′ GATAACAGGCAAGCTTTTGAGG 3′ R: 5′ CTGCAAAAGATTGTTTGGCAGA 3′	59 °C	[57]
*vacA*s	256 (s1)/286 (s2)	F: 5′ ATGGAAATACAACAAACACAC 3′ R: 5′ CTGCTTGAATGCGCCAAAC 3′	58 °C	[57]
*vacA*m	570 (m1)/645 (m2)	F: 5′ CAATCTGTCCAATCAAGCGAG 3′ R: 5′ GCGTCTAAATAATTCCAAGG 3′	56 °C	[57]

**Table 2:** Primer sets used for estimating the expression of toxin genes of *H. pylori* by RT-qPCR.

Target Gene	Sequences	References
*cagA*	F: 5′ TTGACCAACAACCACAAACCGAAG 3′ R: 5′ CTTCCCTTAATTGCGAGATTCC 3′	[58]
*vacA*	F: 5′ GGTCAAAATGCGGTCATGG 3′ R: 5′ CCATTGGTACCTGTAGAAAC 3′	[58]
*16S rRNA*	F: 5′ CTCATTGCGAAGGCGACCT 3′ R: 5′ TCTAATCCTGTTTGCTCCCCA 3′	[59]

## Data Availability

Data are contained within the article. Further inquiries can be directed to the corresponding author.

## Supplementary Materials

The following supporting information can be downloaded at: https://www.mdpi.com/article/10.3390/ijms262110399/s1.

## Abbreviations

BHI—Brain-Heart Infusion; CagA—cytotoxin gene A; cDNA—complementary DNA; CLR—clarithromycin; DMSO—dimethyl sulfoxide; EVs—extracellular vesicles; FBS—fetal bovine serum; FCS—fetal calf serum; IL-8—interleukin 8; LEV—levofloxacin; MIC—minimal inhibitory concentration; MTZ—metronidazole; NTA—nanoparticle tracking analysis; PCR—polymerase chain reaction; RT-qPCR—reversed transcription-quantitative polymerase chain reaction; T4SS—type 4 secretion system; T5SS—type 5 secretion system; TEM—transmission electron microscopy; TSB—Tryptic Soy Broth; VacA—vacuolating cytotoxin A.
