# Supplementary material for: Antibiotics Impact the Cytotoxicity and Cytopathic Effect of Helicobacter pylori Extracellular Vesicles Against Gastric Cells
Source: Int J Mol Sci. 2025 Oct 26;26(21):10399. doi: 10.3390/ijms262110399 (PMC12607952; doi:10.3390/ijms262110399)
Supplement: Supplementary file 1 [file ijms-26-10399-s001.zip › ijms-3895738-supplementary.pdf]

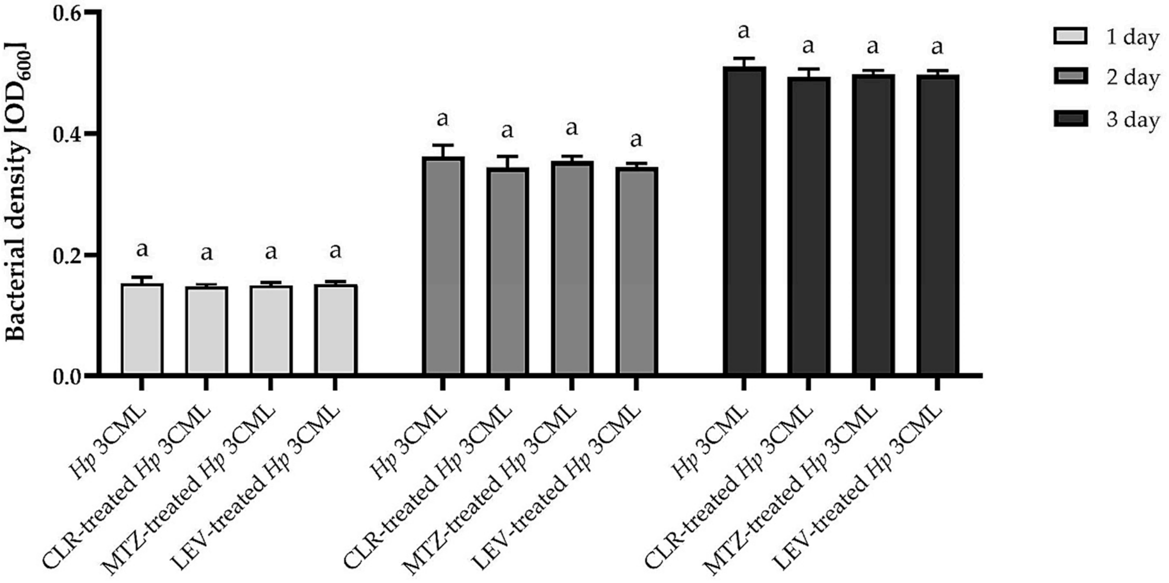

Figure S1. The effect of subinhibitory antibiotic concentrations on the cell density of *H. pylori* 3CML. Bacteria were cultured in 24-well titration plates filled with BHI + 10% FCS^{-EVs} for 1 – 3 days at 37 °C, microaerophilic conditions and were shaked at 100 rpm. The density of bacteria was measured spectrophotometrically (OD_{600}) in three biological replications (n = 3). Values with the same letters in columns are not statistically different ($p > 0.05$). Legend: clarithromycin (CLR), metronidazole (MTZ), levofloxacin (LEV).
